# Derivation of Functional Human Astrocytes from Cerebral Organoids

**DOI:** 10.1038/srep45091

**Published:** 2017-03-27

**Authors:** Rômulo Sperduto Dezonne, Rafaela Costa Sartore, Juliana Minardi Nascimento, Verônica M. Saia-Cereda, Luciana Ferreira Romão, Soniza Vieira Alves-Leon, Jorge Marcondes de Souza, Daniel Martins-de-Souza, Stevens Kastrup Rehen, Flávia Carvalho Alcantara Gomes

**Affiliations:** 1 Instituto de Ciências Biomédicas, Universidade Federal do Rio de Janeiro, Rio de Janeiro, RJ., Brasil; 2 Instituto D’Or de Pesquisa e Ensino (IDOR), Rio de Janeiro, RJ, Brasil; 3 Instituto de Biologia, Universidade Estadual de Campinas, Campinas, SP, Brasil; 4 Universidade Federal do Rio de Janeiro,Campus Xerém, RJ, Brasil; 5 Hospital Universitário Clementino Fraga Filho, Universidade Federal do Rio de Janeiro, Rio de Janeiro, RJ, Brasil.

## Abstract

Astrocytes play a critical role in the development and homeostasis of the central nervous system (CNS). Astrocyte dysfunction results in several neurological and degenerative diseases. However, a major challenge to our understanding of astrocyte physiology and pathology is the restriction of studies to animal models, human post-mortem brain tissues, or samples obtained from invasive surgical procedures. Here, we report a protocol to generate human functional astrocytes from cerebral organoids derived from human pluripotent stem cells. The cellular isolation of cerebral organoids yielded cells that were morphologically and functionally like astrocytes. Immunolabelling and proteomic assays revealed that human organoid-derived astrocytes express the main astrocytic molecular markers, including glutamate transporters, specific enzymes and cytoskeletal proteins. We found that organoid-derived astrocytes strongly supported neuronal survival and neurite outgrowth and responded to ATP through transient calcium wave elevations, which are hallmarks of astrocyte physiology. Additionally, these astrocytes presented similar functional pathways to those isolated from adult human cortex by surgical procedures. This is the first study to provide proteomic and functional analyses of astrocytes isolated from human cerebral organoids. The isolation of these astrocytes holds great potential for the investigation of developmental and evolutionary features of the human brain and provides a useful approach to drug screening and neurodegenerative disease modelling.

Astrocytes have been the subject of a huge paradigm shift in neurosciences during the last decades. Initially classified as passive and merely supporting cells, today, astrocytes are known as active components of brain development, homeostasis and function. These features include the regulation of blood flow, blood brain barrier formation and function, and synapse formation and elimination^1^,^2^,^3^,^4^. Additionally, astrocytes play a role in the control of neurotransmitters and potassium levels and as a source of neurotrophic and survival factors^5^,^6^,^7^,^8^,^9^,^10^,^11^,^12^.

Due to its overall role in brain function, astroglial dysfunction has been implicated in most neurological disorders and neurodegenerative diseases. However, our understanding of astrocyte physiology and pathology has been restricted to animal models, human postmortem brain tissue and astrocytes derived from clinical invasive procedures. Additionally, recent observations have revealed that murine and human astrocytes differ considerably not only morphologically but also functionally and at the molecular level^13^,^14^,^15^,^16^. These data highlight the need for the development of non-invasive experimental models for studying human brain astrocytes.

Several groups have focused on deriving astroglia from rodent and human progenitor cells (for a review, see Chen *et al*., 2015). A few caveats of the methods utilized to date include long-term culture, invasive surgical procedures for obtaining human cells, questionable cell culture purity, and the use of specific induction factors, such as fibroblast growth factor (FGF), leukemia-inhibiting factor (LIF), ciliary neurotrophic factor (CNTF) or bone morphogenetic proteins (BMPs), which not only endure the procedure but drive progenitors towards a specific astrocyte phenotype.

Recently, organoids derived from human stem cells such as human embryonic stem cells (ESCs), tissue-specific progenitor cells or induced pluripotent stem cells (iPSs) have emerged as an appropriate model for the study of human development, since they spontaneously recapitulate the morphogenic features and functions of living organs *in vitro*^17^,^18^.

Cerebral organoid generation is based on the intrinsic capacity of human pluripotent stem cells to differentiate towards neural lineages without an external supply of morphogens or pharmacological inhibitors^18^,^19^,^20^. Recently, by using an adapted protocol from Lancaster and co-worker, we developed a new approach for the production of cerebral organoids in spinner flasks^21^. These brain organoids resemble the human cerebral cortex, as evidenced by the presence of radial glia progenitor cells, ventricular and subventricular zones, intermediate progenitors and an organized cortical plate^19^,^20^,^22^.

Here, we report the development of a simple and efficient protocol for generating purified functional cortical human astrocytes from cerebral organoids. The astrocytes were isolated from cerebral organoids derived from ESCs. All derived cells presented a morphology and molecular markers compatible with human cultured astrocytes. Moreover, functional assays attested their astrocytic phenotype, including a transient elevation of calcium waves in response to ATP and neurotrophic properties. Our work provides a simple and reproducible method that holds the potential to reveal human astrocytes behaviour in neuropsychiatric and degenerative disorders, address comparative evolutionary studies and might provide a suitable tool for drug screening and disease modelling *in vitro*.

## Results

### Astrocyte isolation from cerebral organoids and morphological characterization

**Figure 1:**
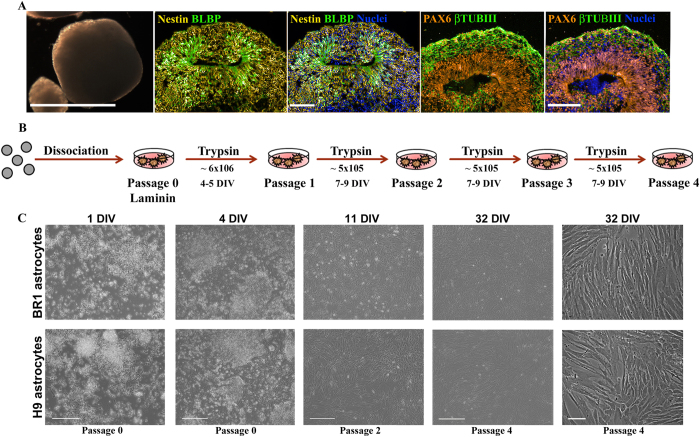
*Astrocyte isolation protocol*. (**A**) Cerebral organoids were maintained for 45 days, followed by immunostaining for Nestin, BLBP, β-tubulin III and PAX6. Progenitor cell bodies (radial glial cells) were observed around a cavity resembling the ventricle and cortical plate and the progenitor zones stained with β-tubulin III and PAX6, respectively. (**B**) For astrocyte isolation, cerebral organoids were mechanically dissociated and cells were plated onto a laminin-coated 100-mm culture dish (Passage 0). After 4 days at passage 0, the cells were enzymatically removed by trypsin digestion, plated in a 100-mm culture dish, and maintained until reaching confluency (7–9 DIV, approximately) (Passage 1). This step was successively repeated to achieve Passages 2, 3, and 4. All experiments described herein were conducted at Passage 4. (**C**) Cells were isolated from H9 and BR1 cerebral organoids at 1 DIV (Passage 0), 4 DIV (Passage 0), 11 DIV (Passage 2) and 32 DIV (Passage 4). Note that the cells present a flat, fibroblast-like appearance, which is characteristic of astrocytes in culture (11 and 32 DIV). Scale bars: 1000 μm cerebral organoid light microscopy (**A**); 100 μm fluorescence microscopy (**A**); 200 μm (C, 1 and 4 DIV); 500 μm (C, 11 and 32 DIV); 100 μm (C, 32 DIV).

Cerebral organoids were derived from embryonic stem cells (BR1 and H9) as previously described^19^,^20^. After 45 days of differentiation (Fig. 1A), they presented a well-organized structure composed of radial glia (RG) cells characterized by Nestin filament that spanned all organoids and BLBP (brain lipid binding protein) located around cavities that resemble cerebral ventricles (Fig. 1A). Cerebral organoids also presented staining for β-tubulin III in a well-organized cortical plate, and staining for the progenitor marker, Pax6, around ventricle-like structures that establish the ventricular and subventricular zones (Fig. 1A). At this stage, approximately 15 cerebral organoids from each lineage were mechanically dissociated, and the cells were plated onto a 100-mm culture dish that was previously coated with polyornithine and laminin to enhance neuronal progenitor adhesion; this was assigned as passage 0 in the scheme (Fig. 1B). To enrich the pool of astrocyte progenitors, the cells were cultured in astrocyte basic growth medium consisting of DMEM-F12 supplemented with 10% FBS for 4 days *in vitro* (DIV)^7^,^10^,^23^,^24^. After 24 hours, a heterogeneous culture was obtained that contained undissociated cells from the cerebral organoid, and astrocyte-like cell (Fig. 1C, 1 DIV). At 4 DIV, we observed an astrocyte-like monolayer underneath neurons and progenitors (Fig. 1C, 4 DIV). Subsequently, the cells were expanded through 4 consecutive passages (Fig. 1B). After the initial passages, the culture became homogeneous (Fig. 1C), with an astrocyte-like morphology (Fig. 1C, 11 and 32 DIV) were obtained. After a month in culture, we performed molecular and functional characterization of astrocytes at passage 4, that is, 32 DIV (Fig. 1C, 32 DIV).

To morphologically define human astrocytes derived from BR1 and H9 cerebral organoids, the cells were analysed by light microscopy. The cells developed a flat, fibroblast-like morphology as previously described for astrocytes maintained *in vitro*. No significant morphological difference was noted between the cell lines (Fig. 1C, 32 DIV). Similarly, the culture expansion was successful, and the growth rate was equivalent between cell lines.

### Molecular characterization

**Figure 2:**
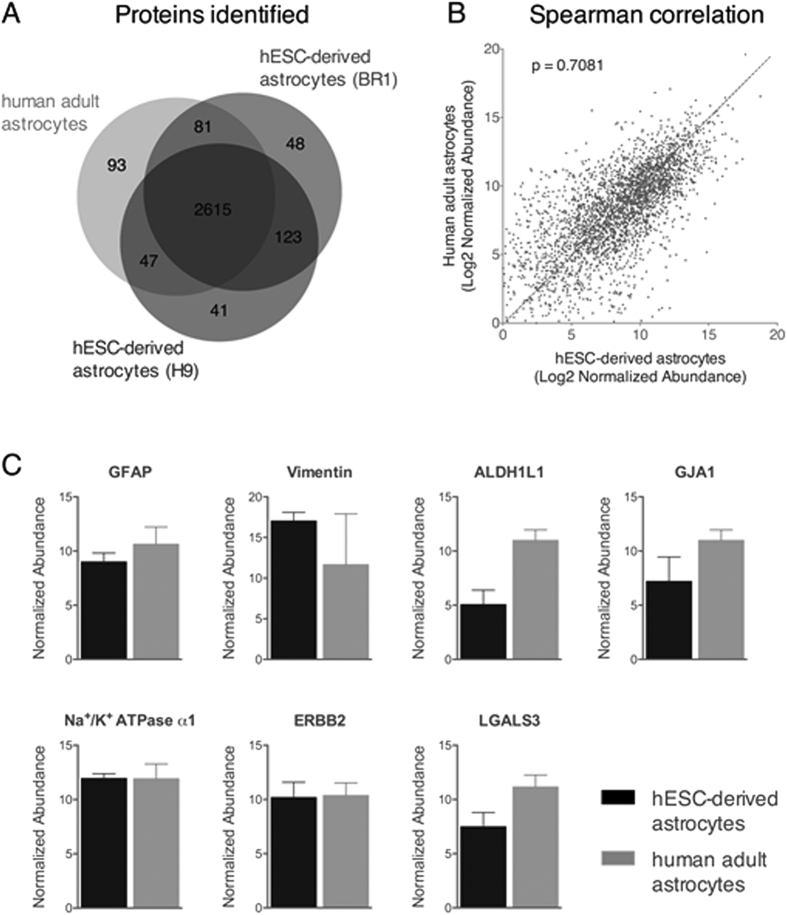
Proteomics profile of astrocytes derived from hESC-cerebral organoids and human adult surgical specimens. (**A**) Venn diagram comparing the number of proteins identified by shotgun mass spectrometry of astrocytes derived from hESC cell lines, BR1 and H9-cerebral organoids, and human adult astrocytes. (**B**) Spearman correlation between global relative protein expression of hESC-cerebral organoids-astrocytes and human adult astrocytes. p represents the spearman correlation coefficient. (**C**) Plot of selected proteins related to astrocytes; the comparison was normalized to the abundance of proteins from hESC-derived and human adult astrocytes.

We subsequently performed a molecular characterization by identifying astrocytic markers by immunocytochemistry and proteomic assays. To evaluate the global protein expression of astrocytes derived from stem cells, we performed state-of-the-art shotgun proteomics and identified 3,048 proteins, demonstrating the overlap of 85.8% of the identified proteins (Fig. 2A) among hESC-derived astrocytes from BR1 and H9, as well as human adult astrocytes. In addition, the Spearman correlation analysis revealed a strong correlation regarding the relative protein abundances between hESC-derived astrocytes and human adult astrocytes (P = 0.7081, Fig. 2B). For example, we show commonly identified proteins in astrocytes, such as GFAP (glial fibrillary acidic protein), vimentin and ALDH1L1 (aldehyde dehydrogenase 1 family member L1), both in hESC-derived and adult astrocytes, with similar abundances (Fig. 2C). We also detected markers of immature astrocytes such as ERBB2 and galectin 3 (LGALS3) in both samples, with no significant differences.

**Figure 3:**
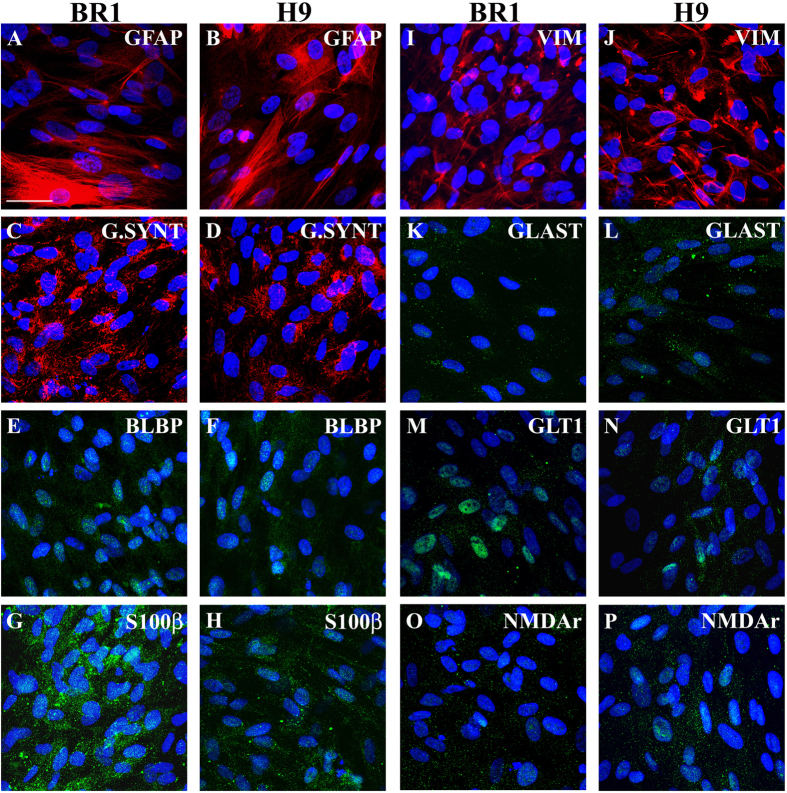
*Morphological and immunostaining characterization of astrocytes derived from BR1- and H9-cerebral organoids*. Immunostaining of astrocytes (Passage 4) derived from BR1/H9-cerebral organoids for astroglial markers after 7 DIV. GFAP **(A,B)**, glutamine synthetase (G.SYNT; **C,D**), brain lipid binding protein (BLBP; **E,F**), S100β (**G,H**), vimentin (**I,J**), glutamate transporter GLAST (**K-L**), glutamate transporter GLT-1 (**M,N**), N-methyl-D-aspartate receptor (NMDAr; **O,P**). Nuclei were stained blue (DAPI). Scale bar: 50 μm.

Immunolabelling assays revealed that all human astrocytes derived from cerebral organoids from both hESC lineages presented most of the main astrocyte makers, including GFAP, glutamine synthetase, BLBP (brain lipid-binding protein), S100β, and vimentin. Additionally, differentiated astrocytes were also positive for the glutamate transporters, GLAST (glutamate aspartate transporter) and GLT1 (glutamate transporter 1), and for the NMDA (N-methyl-D-aspartate) receptor (Fig. 3). The cultures showed no reactivity towards Nestin, olig2 (oligodendrocyte transcription factor), FOXG1 (forkhead box G1) or β-tubulin III (Table 1), attesting their commitment towards the astroglial lineage.

### Protein-protein interaction pathways (interactome)

**Figure 4:**
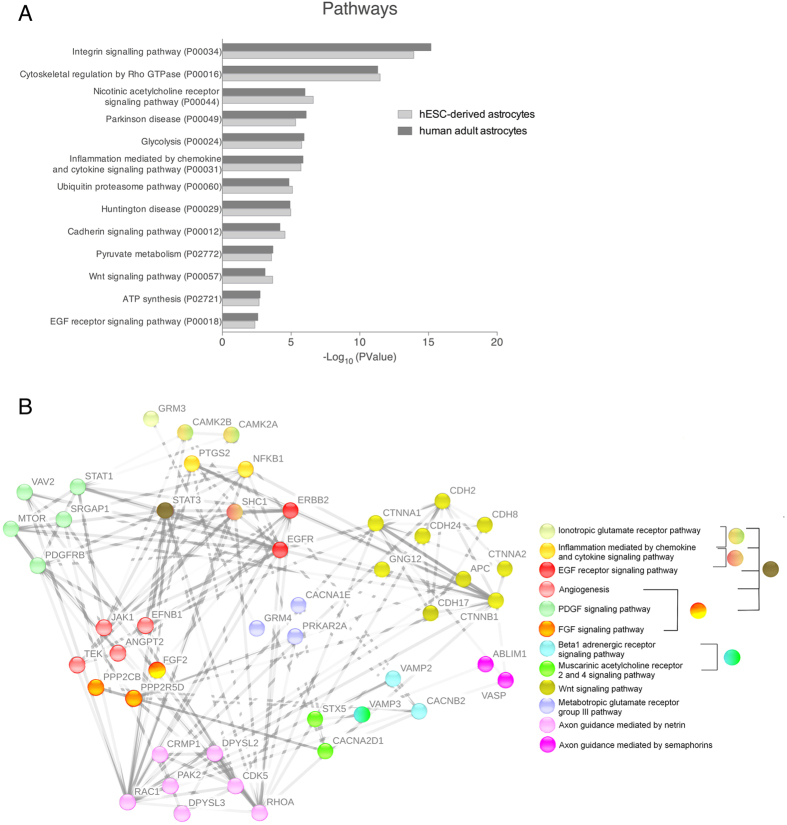
Network interactive organization of proteins from astrocytes. (**A**) Functional enrichment of canonical pathway proteins in astrocytes derived from hESC-cerebral organoids and human adult cortex. (**B**) Interactive network representation of the molecular relationship between proteins found in astrocytes from the dataset based on String (string-db.org/).

We identified over-represented ontological groups and pathways amongst proteins of BR1 and H9 astrocytes compared with human adult astrocytes. As shown in Fig. 4, there were no significant differences between these two groups. BR1 and H9 astrocytes presented the same major thirteen representative pathways observed in adult human astrocytes. Using the specific proteins found in the proteomic assay, we explored the interactome of BR1 and H9 astrocytes. We identified proteins involved in pathways related to axonal guidance, such as ephrins (EFNB1) and netrins (NTN4); angiogenic activity, including angiopoietin-2 (ANGPT2), EFNB1 and bFGF; neurotrophic growth factors, such as bFGF; glutamate receptor response and intracellular calcium effectors, such as CAMK (CAMK2A and CAMK2B); the neuregulin response, EGFR and ERBB2; and the inflammatory-mediated response, NFk-β (NFKB1). As demonstrated in Fig. 4, the interactome of astrocytes derived from BR1 and H9 embryonic cell lines is very similar to those isolated from adult human cortex.

### Functional characterization

**Figure 5:**
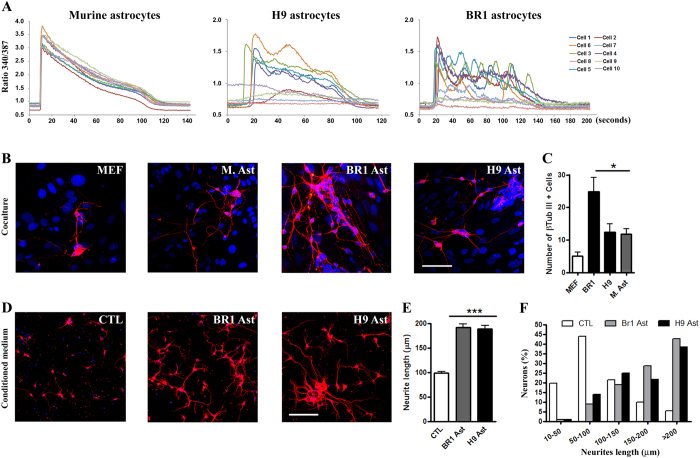
Functional characterization of BR1 and H9-cerebral organoids derived astrocytes. (**A**) Intracellular calcium levels [Ca^2+^] were measured after ATP stimulation in three different astrocyte monolayers: neonatal secondary cortical murine astrocytes, H9-derived astrocytes, and BR1-derived astrocytes. The graphs show the internal variations in [Ca^2+^]_i_ over time (seconds). The astrocyte cultures were loaded for 40 min with Fura-2/AM and pluronic acid F-127 in Krebs solution. The cells were continuously perfused with Krebs solution and stimulated with 100 μM ATP (at 5 s) for 60 s. (**B**) Embryonic murine cortical neurons were cultured over four different astrocyte monolayers: murine embryonic fibroblasts (MEFs); neonatal secondary cortical murine astrocytes (M. Ast); BR1 astrocytes and H9 astrocytes. After 48 h of coculture, the cells were stained for β-tubulin III, and the number of neurons was quantified (**C**). **(D)** Embryonic murine cortical neurons were cultured in the presence of different conditioned media, and the neurite length was measured after 48 h: BR1 astrocyte conditioned medium, H9 astrocyte conditioned medium and neurobasal medium (Control). At least 100 neurons were randomly chosen and analysed in each condition; (**E**) neurite length; **(F)** neurite length distribution in each population. Nuclei stained in blue (DAPI). Scale bars: 50 μm (**B**) and 100 μm (**D**), *P < 0.05; ****P < 0.001; N = 3.

To determine whether astrocytes derived from cerebral organoids were biologically active, we performed two functional assays: analysis of neurotrophic support and measurement of calcium waves in response to ATP stimuli. Mouse and human astrocytes respond to sensory inputs, such as glutamate and ATP, via transient calcium waves^25^,^26^,^27^,^28^. To investigate whether human astrocytes derived from our protocol also respond to these extracellular cues, we performed calcium imaging assays. We treated BR1 and H9-derived astrocytes for 60 s with an influx of 100 μM ATP. An efficient response to ATP was considered 20% above the cellular baseline. As shown in Fig. 5, all murine astrocytes responded to ATP (Fig. 5A). In BR1 (Fig. 5A) and H9 (Fig. 5A) astrocyte cultures, approximately 50% of the cells responded efficiently to ATP. Of note, the responsive cells presented a well-recognized asynchrony, which is a feature of human astrocytes^25^.

Astrocytes constitute the main source of neurotrophic factors during nervous system development^7^,^10^,^24^,^29^,^30^,^31^. To investigate neurotrophic properties from BR1 and H9 astrocytes, we tested whether human astrocytes promote neuronal survival by using a coculture model with murine embryonic cortical neurons. After 48 h, the number of murine β-tubulin III-positive cells was quantified. As a positive control, we used murine cortical neonatal astrocytes, which are known to support neuronal survival. Murine embryonic fibroblasts (MEFs) served as negative controls (Fig. 5B). We found that human astrocytes derived from cerebral organoids strongly sustained neuronal survival (Fig. 5B and C). Astrocytes derived from BR1 organoids presented an even increased supportive capacity compared with their murine counterparts (Fig. 5C). However, MEFs provided poor support for cortical neurons (Fig. 5C), indicating that neuronal survival was not due to cell contact but specifically to neuron-astrocyte signalling. Our data show that human astrocytes derived from BR1 and H9 cerebral organoids share the ability of murine astrocytes to promote neuronal survival *in vitro*.

In due course, to investigate the neurotrophic properties of soluble factors secreted by BR1 and H9 astrocytes, embryonic cerebral cortex progenitors were cultured for 48 hours in the presence of conditioned media obtained from BR1 and H9 astrocytes (BR1 or H9-ACM), or neurobasal medium alone (Fig. 5D). To evaluate the effect of BR1 and H9-ACM on neurite growth, neurite lengths were analysed using three parameters: either considering the sum of the total neurite length per neuron (Fig. 5E), the longest neurite per neuron or the sum of all neurite measurements divided by the number of processes per neuron (data not shown). BR1 and H9 ACM promoted a 100% increment in neurite outgrowth (Fig. 5E). Moreover, approximately 40% of the neuronal cells grown on BR1 and H9-ACM developed neurites with an average size of more than 200 μm, whereas only approximately 5% of the neurons plated on neurobasal (Fig. 5D Control) medium displayed these characteristics (Fig. 5F). Together, these results show that BR1 and H9 astrocytes are neurotrophic and induce cortical progenitor neurite outgrowth.

The data presented herein show that astrocytes derived from BR1 and H9 organoids present morphologic, genetic and functional properties that confirm their astrocytic identity. Together, our data show that the protocol described herein is useful for the large-scale isolation of functional human astrocytes from two different lines, BR1 and H9.

## Discussion

Here, we present a new protocol to isolate human astrocytes from cerebral cortical organoids. We demonstrate that human astrocytes derived from BR1 and H9 cerebral organoids develop a flat, fibroblast-like appearance as expected for cultured human astrocytes^22^. Our molecular portrayal, by immunolabelling and proteomic assays, revealed that cells derived from both lineages present most characteristic astrocyte markers. Functional experiments validated that both BR1 and H9 astrocytes are supportive of cortical murine neurons and induce neurite outgrowth. Furthermore, at least 50% of BR1 and H9 astrocytes also response to an ATP stimulus with asynchronous calcium waves^25^, a hallmark of the biological function of astrocytes. We suggest that our method might provide a useful new tool for drug screening and neurodegenerative disease modelling.

Other groups have succeeded in establishing standard protocols for the generation of astrocytes from ESC and iPS by supplementation with different growth factors such as cardiotrophin 1 (CT-1), LIF or other gliogenic molecules^11^,^32^,^33^,^34^,^35^. The methods used to date present several constraints including long-term culture and a questionable culture purity that could eventually drive the cells towards a specific phenotype due to the use of differentiation factors and the high cost of the procedure.

Here, we developed a time-saving protocol of astrocyte differentiation and isolation based on FBS medium supplementation, followed by subsequent passaging of progenitor cells isolated from cortical organoids. Each 100-mm dish was divided into five or more dishes during these passages, which allowed greater expansion of isolated cells. Additionally, previous works only examined the morphology of GFAP + cells after cerebral organoid dissociation, with maintenance in defined serum-free medium^22^. This is the first description of cerebral organoids as a useful resource for the isolation of functional astrocytes, which not only respond to ATP signalling through calcium waves but sustain neuronal survival and induce neurite outgrowth of cortical murine embryonic neurons.

BR1 and H9 astrocytes developed a flat, fibroblast-like appearance, as expected for astrocytes cultured in the presence of FBS^22^. Likewise, cells express some of the major astrocyte markers, such as GFAP, S100β, glutamine synthetase, and connexin 43^36^,^37^,^38^,^39^, indicating a high specificity towards astroglial commitment. Additionally, non-astrocyte markers, including the progenitor markers Nestin and FOXG1, markers of progenitor cells (radial glia)^40^,^41^,^42^,^43^, were not identified in these cells, thus indicating a differentiation/maturation process. Additional characteristic markers of cultured astrocytes were present, such as vimentin, BLBP and GLAST^40^,^44^,^45^,^46^,^47^. These later findings might also indicate the reactivity that was previously reported to be induced by FBS^22^. It is well-known that reactive astrocytes develop some characteristics of immature cells, such as the expression of vimentin and BLBP^48^,^49^,^50^,^51^,^52^,^53^,^54^,^55^,^56^. In addition, BR1 and H9 astrocytes stained for rNMDA and GLT1, which are specific for mature astrocytes^57^,^58^.

In conclusion, we have identified several astrocytic proteins in astrocyte derived from organoids, including metabolic enzymes, cytoskeleton proteins, calcium binding protein, neurotransmitters receptors. It is important to note that although cells express different intensity of staining, they all stain for all astrocytic markers (double and triple staining), attesting their astrocytic phenotype.

The heterogeneity of astrocytes phenotypes obtained here is an important issue and little is known about the mechanisms that direct astrocyte diversity and whether heterogeneity is represented *in vitro*. Besides, there are no well-established regional markers of astrocytes that could distinguish subpopulations of astrocytes either *in vitro* or *in vivo*. Recently, our group have identified different profile of synaptogenic molecules produced by astrocytes from different regions^59^. The presence of forebrain marker, FOXG1 in the brain organoids, suggest that cells derived from those organoids might present forebrain characteristics^60^,^61^.The astrocytes derived here are probably generated by radial glia cells present in a defined progenitor zone staining positive for Nestin and BLBP.

Here, we also evaluated the global protein expression of BR1- and H9-derived astrocytes by performing state-of-the-art shotgun proteomics, and we identified 2,616 common proteins compared with human adult isolated astrocytes. This revealed an 85.8% overlap of identified proteins. In addition, we found a strong correlation regarding the relative abundances of those proteins with human adult astrocytes, as shown by Spearman correlation. Altogether, these data strongly support the similarity between astrocytes derived from BR1 and H9 ESCs to those isolated from adult human cortex. The three astrocytic markers GFAP, ALDH1L1 (aldehyde dehydrogenase 1 family member L1) and GJA1 (gap junction protein alpha 1), previously described by Zhang and co-workers^25^, showed similar levels in ESC-derived astrocytes and those isolated from human adults. Both samples also presented equal levels of markers expressed by immature astrocytes like vimentin^51^, ERBB2^62^,^63^,^64^ and galectin 3 (LGALS3)^25^. Together, our data show that astrocytes derived from human organoids present combined features of immature and mature human astrocytes.

Non-invasive isolation of astrocytes from humans might represent a powerful tool for modelling neurological diseases. To ensure that our protocol provides an efficient method for the derivation of human astrocytes from individuals, we have also applied the above-described protocol to cerebral organoids derived from iPS obtained from fibroblasts^65^(data not shown). In early passage 2, astrocytes obtained from iPS-organoids previously expressing astroglial markers confirmed that our protocol could successfully induce astrocyte commitment and isolation from organoids derived either from human embryonic stem cells or induced pluripotent stem cells (data not shown).

A hallmark of astroglial physiology is the ability to respond to ATP or glutamate by evoking calcium waves to control synaptic and neural circuit formation and function^2^,^16^,^25^,^66^,^67^,^68^,^69^. We demonstrated herein that both BR1 and H9 astrocytes respond to 100 μM ATP influx with calcium waves. However, 50% of the cells in each culture were capable of eliciting this proper answer, in contrast to the murine samples (100% response). The observed asynchronous pattern was expected for human astrocytes, as previously described by Zhang and coworkers^25^. Such differences in calcium wave oscillation might represent one of the differences between astrocytes from distinct species and might underlie the biological functional complexity of human astrocytes in comparison to their rodent counterparts^13^,^14^,^15^. As previously described all cells express GFAP and several other astrocytic markers (100%), although with different intensity. Based in these observations, we suggest that cells derived from organoids are all astrocytes. However, differences in astrocyte markers staining and calcium waves might be attributed either to different subpopulation of astrocytes or to different developmental stages of these cells. Different levels of GFAP expression have been reported either for different brain regions and for subpopulations in the same region^59^. The presence of astrocytes in different maturation stages is also supported by the fact that astrocytes maintained for 30 days *in vitro* respond more robustly to ATP, in cell number, (70% BR1 and 63,8% H9) compared with those from 7 days (50%), suggesting that those cells mature *in vitro*.

In the CNS, astrocytes are the major source of trophic factors and the extracellular matrix that guides neuronal morphogenesis^10^,^11^,^30^,^70^,^71^,^72^. To confirm that the BR1 and H9-derived astrocytes were biologically functional cells, we performed two neurotrophic support assays: neuronal support and axonal growth assays. BR and H9 astrocyte monolayers provided a good substrate for neuronal survival. However, in BR1 and H9 human astrocyte monolayers, murine neurons were preferentially arranged into islands, probably due to some specificity between species. We also showed that conditioned media from BR1 and H9 derived astrocytes increased the neurite outgrowth of cortical murine embryonic neurons.

Astrocyte conditioned medium contains soluble factors with neurotrophic properties. We identified over-represented ontological groups and pathways amongst proteins of BR1 and H9 astrocytes compared with human adult astrocytes. Using proteomic and bioinformatic assays, we identified some proteins related to axonal guidance like ephrins and netrins^73^, as well as neurotrophic growth factors such as bFGF^74^. A complete analysis of the secretome profile would provide further information about the growth factors that are secreted by human astrocytes. The interactome might shed light into the biological function of astrocytes since the proteins listed are representative of enriched pathways and interactions between them. Among these pathways, we also detected potentially functional pathways represented by the intracellular calcium effector, CAMK^75^; the inflammatory mediated response marker, NFk-β^76^; and molecules related to angiogenic activity, such as angiopoietin-2^77^, EphrinB1 and bFGF^78^. The proteins found on these well-known pathways do not differ compared with adult surgery isolated astrocytes, validating the similarities between them.

Our work provides a useful tool to study human astrocyte physiology independently of conventional animal models. This is especially important because recent observations revealed that murine and human astrocytes differ morphologically and functionally at the molecular level^13^,^14^,^15^,^16^. In contrast to post-mortem brain tissue or astrocytes obtained by surgery, the method described herein offers a simple, non-invasive procedure to access human astrocytes. This is the first study to provide proteomic and functional analyses of astrocytes isolated from cerebral organoids. The protocol described herein might represent a useful model for studying the roles of human astrocytes in brain function and their implications for neuropsychiatric and degenerative disorders.

## Materials and Methods

### Ethical approval

All animal protocols were approved by the Animal Research Committee of the Federal University of Rio de Janeiro (CEUA01200.001568/2013-87). Procedures were all in accordance with the ‘Guide for the Care and Use of Laboratory Animals’. Efforts were made to minimize animal suffering and to reduce the number of animals used. Human adult astrocytes were isolated from patients selected for surgical treatment of temporal lobe epilepsy associated with hippocampus sclerosis at the Hospital Universitário Clementino Fraga Filho All patients provided written consent to participate in the study, and the procedures were in agreement with the Brazilian Ministry of Health Ethics Committee under the protocol CONEP2340.

### Murine astrocyte secondary cultures

Astrocyte primary cultures were prepared from cerebral cortex derived from new-born Swiss mice as previously described^79^. Briefly, after decapitation, mouse brain structures were removed, and the meninges were carefully stripped off. Tissues were washed in phosphate-buffered saline (PBS) containing 0.6% glucose (Merck, Darmstadt, Hessen, DE), and cortical structures were dissociated into single cells in a medium consisting of Dulbecco’s Modified Eagle’s Medium supplemented with the nutrient mixture F-12 (DMEM/F-12, Invitrogen Life Technologies, Carlsbad, California, USA) and enriched with glucose (3.3 × 10^−2^ M), glutamine (2 × 10^−3^ M) and sodium bicarbonate (0.3 × 10^−2^ M). Dissociated cells were plated onto plastic culture flasks or glass coverslips (24-well plates, Techno Plastic Products, Trasadingen, CH) that were previously coated with polyornithine (1.5 μg/mL, molecular weight 41,000; Sigma Chemical Co., St Louis, Missouri, USA) in DMEM/F12 supplemented with 10% foetal bovine serum (FBS) (Invitrogen). The cultures were incubated at 37 °C in a humidified 5% CO_2_, 95% air chamber. After 24 h, the cell cultures were washed, and the medium was replaced with DMEM/F-12 supplemented with 10% FBS. The medium was changed every second day until the cells reached confluence, at which time the astrocyte monolayers were enzymatically dissociated with trypsin (Sigma) for 5 min at 37 °C in a humidified 5% CO_2_, 95% air chamber. Trypsin was inhibited by addition of DMEM-F12 supplemented with 10% FBS and centrifuged (~200 g) for 5 min. The cells (10 × 10^5^) were plated on glass coverslips that had been previously coated with polyornithine (1.5 μg/mL; Sigma), and the cultures were incubated at 37 °C in a humidified 5% CO_2_, 95% air chamber in DMEM/F-12 supplemented with 10% FBS. The medium was changed every second day until the cells reached confluence.

### Human adult astrocyte isolation and culture

Adult primary human astrocytes were isolated from surgically resected anterior temporal lobe tissue and from patients who were selected for surgical treatment of temporal lobe epilepsy associated with hippocampus sclerosis as previously described^3^. The selected patients were evaluated by video-electroencephalography monitoring with a 132-channel Nihon-Kohden^®^apparatus, and the ictal onset zone was concordant with neuroimaging and semiology data. The pathological tissue targeted in surgery for these cases is the gliotic hippocampus, and the anterior temporal lobe resection is used merely as a surgical pathway to the diseased area. As described previously, only healthy cortical tissue was used to produce astrocyte cultures. Experimental protocols were performed as described previously^37^. Briefly, the tissues were washed in DMEM-F12 (Invitrogen), mechanically dissociated, chopped into small (<2 mm^3^) pieces with a sterile scalpel, and incubated in 10 mL of 0.25% trypsin solution at 37 °C for 10 min. After centrifugation for 10 min, the cell pellet was suspended in DMEM/F-12 growth medium supplemented with 10% FBS (Invitrogen) and plated on tissue culture plates in a humidified 5% CO_2_, 95% air atmosphere at 37 °C for 2 h to allow the microglial cell to adhere. The nonadherent astrocytes were transferred into other culture plates that had been coated with polyornithine (1.5 μg/mL; Sigma). Adherent astrocytes were allowed to grow by replacing the medium once a week. New passages of cells were generated by harvesting confluent astrocyte cultures using trypsin (Sigma). Human astrocytes collected from up to the third passage were used in this study.

### Cerebral organoid production

Human embryonic stem cells (hESC), BR-1^80^ and H9^81^ cell lines were cultured in mTeSR1 medium (Stemcell Technologies, Vancouver, Canada) on a Matrigel (BD Biosciences)-coated surface. The colonies were manually passaged every seven days and maintained at 37 °C in humidified air with 5% CO_2_. The differentiation into cerebral organoids was performed as previously described^21^. Briefly, 250,000 cells/mL were inoculated into a spinner flask containing mTeSR1 medium supplemented with 10 μM Y-27632 (Rho-associated protein kinase inhibitor, iRock) (Merck) under uninterrupted exposure to 40 rpm. After 24 h, the medium was replaced with embryoid body media. By day 6, embryoid bodies were fed neural induction medium containing N2 supplement and heparin. On day 11, cellular aggregates were covered in Matrigel and cultured in differentiation medium containing Neurobasal (Invitrogen), N_2_ (Invitrogen), B27 minus vitamin A (Invitrogen), and insulin. After 4 days, the medium was changed using the same formulation, except with the replacement of B27 with vitamin A (Invitrogen). The medium was changed every week. The cerebral organoids were allowed to grow for 45 days.

### Human astrocyte derivation from cerebral organoids

Cerebral organoids that had been differentiated for 45 days (derived from BR1 and H9 cell lines) were used for astrocyte isolation. Initially, approximately 15 cerebral organoids were mechanically dissociated and plated (~6 × 10^6^ cells/dish) in three 100-mm culture dishes that had been previously coated with polyornithine (1.5 μg/mL, Sigma) and laminin (5 μg/mL in PBS for 4 h, Sigma). They were then maintained in this condition until they reached confluency after approximately four days. The cells were maintained in DMEM/F12 (Invitrogen) supplemented with 10% FBS (Invitrogen). After achieving confluency (passage 0), the cell monolayers were enzymatically dissociated with trypsin (Sigma) for 5 min at 37 °C in a humidified 5% CO_2_, 95% air chamber. Trypsin was inhibited by addition of DMEM-F12 supplemented with 10% FBS followed by centrifugation (~200 × g) for 5 min. The cells (5 × 10^5^) were plated on a 100-mm culture dish that had been previously coated with polyornithine (1.5 μg/mL; Sigma) and grown in DMEM/F-12 supplemented with 10% FBS medium for approximately 7–9 days at 37 °C in a humidified 5% CO_2_, 95% air chamber (passage 1). The medium was changed every second day until the culture reached confluency. The step was repeated successively to acquire passages 2, 3, 4 and 5. For each passage, a cell sample was frozen in 10% dimethyl sulfoxide (DMSO, Sigma) for future use and characterization. Characterizations were performed using passage 4 astrocytes. For functional and immunolabelling assays, astrocytes were maintained (approximately 2 × 10^4^ cells/well) on glass coverslips (24-well plates, Techno Plastic Products, Trasadingen, CH) that had been previously coated with polyornithine (1.5 μg/mL, Sigma) until reaching confluency. Organoids were derived from different series of organoid production (1BR1and 2H9), the number of differentiation experiment performed to obtain enriched astrocytes was successfully achieved in three different times. Individual assays were performed three times, in triplicate, with different pool of cells, before and after freezing.

### Murine embryonic fibroblast (MEF) cultures

Pregnant Swiss females at 2 gestational days were killed by exposure to halothane followed by cervical dislocation, and the embryos (E12) were removed. Head and red organs were dissected and washed in PBS, and all embryos were placed in a clean Petri dish. The embryos were incubated with trypsin (Sigma) for 15 min at 37 °C in a humidified 5% CO_2_, 95% air chamber. After incubation, the embryos were dissociated into single cells, and trypsin was inhibited by addition of DMEM-F12 (Invitrogen) supplemented with 10% FBS (Invitrogen). The sample was centrifuged at 200 g for 5 min, the supernatant was carefully removed and the cell pellet was suspended in warm MEF medium consisting of Dulbecco’s Modified Eagle’s Medium supplemented with the nutrient mixture F-12 (DMEM/F-12, Invitrogen) enriched with glucose (3.3 × 10^−2^ M), glutamine (2 × 10^−3^ M) and sodium bicarbonate (0.3 × 10^−2^ M) and supplemented with 10% FBS (Invitrogen) and 1% of penicillin-streptomycin (Invitrogen). Approximately 5 × 10^5^ cells were plated into 0.2% gelatine-coated flasks (gelatine from bovine skin, Type B, Sigma) for 2 h and incubated at 37 °C in a humidified 5% CO_2_, 95% air chamber in DMEM/F-12 supplemented with 10% FBS medium for 2 h. After a secondary passage, the cells were plated on glass coverslips that had been previously coated with polyornithine (1.5 μg/mL; Sigma). The cultures were then incubated under the same conditions until reaching confluency, and the medium was changed every 2 days.

### Astrocyte conditioned medium preparation

After reaching confluency, the astrocyte monolayers were washed three times with PBS and incubated for two days in neurobasal medium (Invitrogen). After this period, BR1 and H9 astrocyte conditioned media were recovered, centrifuged at 1500 × g for 10 min, and used immediately or stored at −70 °C for further use.

### Neuronal-astrocyte conditioned medium assay

Pregnant Swiss females at 14 gestational days were killed by exposure to halothane followed by cervical dislocation, and the embryos (E14) were removed. Cortical progenitors were prepared as previously described^29^. Briefly, the cells were freshly dissociated from the cerebral cortex, and 10 × 10^4^ cells were plated onto glass coverslips that had been previously coated with polyornithine (1.5 μg/mL; Sigma) and grown in BR1 and H9 astrocyte conditioned medium or neurobasal medium (Invitrogen) for approximately 48 hours at 37 °C in a humidified 5% CO_2_, 95% air chamber.

### Neuron-astrocyte coculture assay

Pregnant Swiss females at 14 gestational days were killed by exposure to halothane followed by cervical dislocation, and the embryos (E14) were removed. Cortical progenitors were prepared as previously described^29^. Briefly, for the coculture assays, the cells were freshly dissociated from cerebral cortex, and 5 × 10^4^ cells were plated onto cell carpets from BR1-derived astrocytes, H9-derived astrocytes, MEFs derived from Swiss mice and murine astrocytes. The cocultures were maintained for 48 hours at 37 °C in a humidified 5% CO_2_, 95% air atmosphere.

### Calcium signalling assay

Variations in free intracellular calcium levels [Ca^2+^]_i_ were evaluated in single cells obtained from human astrocytes at stage 4 isolated from cerebral organoids. The astrocytes were grown as previously described on coated glass coverslips (15 mm) for 7–9 days to allow the formation of a monolayer. As controls, we used passage 1 murine neonatal cortical astrocytes that had been grown for 7–8 days. Astrocyte cultures were loaded for 40 min with 5 μM Fura-2/AM (Molecular Probes, Invitrogen Life Technologies, Carlsbad, California, USA) and 0.02% pluronic acid F-127 (Molecular Probes) in Krebs solution (132 mM NaCl, 4 mM KCl, 1.4 mM MgCl_2_, 2.5 mM CaCl_2_, 6 mM glucose, 10 mM HEPES, pH 7.4) and incubated at 37 °C in a humidified 5% CO_2_, 95% air atmosphere. After the loading period, the cultures were washed three times with Krebs solution to remove the excess probes. The glass coverslip with the cell monolayer was mounted on an RC-20 chamber in a PH3 platform (Warner Instruments, Hamden, CT) and visualized on an inverted fluorescence microscope (Axiovert 200; Carl Zeiss). Cells (approximately 100 cells per field) were continuously perfused with Krebs solution and stimulated with 100 μM ATP (Sigma) solution (diluted in Krebs) for one minute. The solutions were added to the cells via a fast-pressurized (95% air, 5% CO_2_ atmosphere) system (AutoMate Scientific, Inc., Berkeley, CA). The variations in [Ca^2+^]_i_ were evaluated by quantifying the ratio of the fluorescence emitted at 510 nm following alternate excitation (750 ms) at 340 and 380 nm using a Lambda DG4 apparatus (Sutter Instrument, Novato, CA) and a 510-nm long-pass filter (Carl Zeiss) before fluorescence acquisition with a 409 objective and a CoolSNAP digital camera (Roper Scientific, Trenton, NJ). Acquired values were processed using the MetaFluor software (Universal Imaging Corp., West Chester, PA). Values for the Fura-2 fluorescence ratio were calculated based on a cut-off of a 20% increase in the [Ca^2+^] level induced by the stimulus, resulting in the selection of 3 high values after stimulating each cell. At the cell baseline, we chose 10 prior values before ATP injection.

### Immunohistochemistry of cerebral organoids

Cerebral organoids were fixed in 4% paraformaldehyde and incubated with sucrose gradient solutions (10, 20 and 30% in PBS) for 15 min each. They were then embedded in optimal cutting temperature compound (OCT) and frozen in liquid nitrogen. Twenty-micron-thick sections were permeabilized with 0.3% Triton-X (Vetec Química Fina Ltda, Rio de Janeiro, Rio de Janeiro, BR) for 5 min at room temperature. Subsequently, the tissue sections were blocked with 3% bovine serum albumin (BSA, Sigma) and 5% normal goat serum (NGS, Invitrogen) in PBS (block solution) for 1 h and incubated overnight at 4 °C with the specified primary antibody diluted in blocking solution. The primary antibodies used were mouse anti-Nestin (1:200; Merck), rabbit anti-BLBP (1:200; Millipore), and rabbit anti-FOXG1 (1:00; Santa Cruz Biotechnology, Inc.). Secondary antibodies were Alexa Fluor 546 goat anti-mouse (1:1,000; Molecular Probes) and Alexa Fluor 488 goat anti-rabbit (1:400; Molecular Probes). Negative controls were obtained by omitting the primary antibodies; in all cases, no reactivity was observed. DAPI (4′,6-diamidino-2-phenylindole, 1 mg/mL, Sigma) was used to stain nuclei. Images were acquired using an Operetta Imaging System (Perkin Elmer Inc.).

### Immunocytochemistry of astrocytes

Cells were fixed with 4% paraformaldehyde for 15 min and permeabilized with 0.2% Triton-X (Vetec Química Fina Ltda) for 5 min at room temperature. Subsequently, the cells were blocked with 3% BSA (Sigma) and 5% NGS (Invitrogen) in PBS (block solution) for 1 h and incubated overnight at 4 °C with the specified primary antibody diluted in block solution. The primary antibodies were mouse anti-β-tubulin III (1:1,000; Promega Corporation; Madison, Wisconsin, USA), rabbit anti-GFAP (1:500; Dako, Glostrup, DK), rabbit anti-BLBP (1:200; Millipore), mouse anti-glutamine synthetase (1:200; Millipore), rabbit anti-S100β (1:200; Dako), mouse anti-vimentin (1:100; ABCAM), rabbit anti-GLAST (1:200; ABCAM), rabbit anti-GLT1 (1:200; ABCAM), rabbit anti-NMDAr (1:500; ABCAM), mouse anti-Nestin (1:100; Millipore), rabbit anti-olig2 (1:200; ABCAM), rabbit anti-FOXG1 (1:200; ABCAM), mouse anti-Cx43 (1:100; Invitrogen), and rabbit anti-pCx43 (1:500; Sigma). After incubation with the primary antibody, the cells were washed extensively with PBS and incubated with the following secondary antibodies diluted in block solution for 2 h: goat anti-mouse IgG conjugated to Alexa Fluor 546 and 488 (Molecular Probes; 1:1,000 and 1:400, respectively) or goat anti-rabbit IgG conjugated to Alexa Fluor 488 and 546 (Molecular Probes; 1:400 and 1:1,000, respectively). Cell nuclei were labelled with 4′,6-diamidino-2-phenylindole dihydrochloride (DAPI; Sigma), and cell preparations were mounted directly onto Dako faramount aqueous mounting medium (Dako). Negative controls were obtained by omitting primary antibodies; in all cases, no reactivity was observed. After immunostaining, cell cultures were visualized using a Leica SP5 confocal microscope or TE300 Nikon microscope.

### Liquid chromatography-mass spectrometry

Qualitative and quantitative proteomic analyses were performed in a bidimensional micro UPLC tandem nanoESI-HDMSE platform by multiplexed data-independent acquisition (DIA) experiments^82^. The Acquity UPLC M-Class System (Waters Corporation, Milford, MA) coupled to a Synapt G2-Si mass spectrometer (Waters Corporation) was used.

Peptide loads were separated in a nanoACQUITY UPLC HSS T3 Column (1.8 μm, 75 μm X 150 mm, Waters Corporation). Peptides were eluted using an acetonitrile gradient ranging from 7% to 40% (v/v) for 95 min at a flow rate of 0.4 μL/min directly into a Synapt G2-Si. For every measurement, the mass spectrometer was operated in resolution mode with an m/z resolving power of approximately 40 000 FWHM and using ion mobility with a cross-section resolving power at least 40 Ω/ΔΩ. The effective resolution obtained with the conjoined ion mobility was 1 800 000 FWHM. MS/MS analyses were performed by nanoelectrospray ionization in positive ion mode nanoESI (+) and with a NanoLock Spray (Waters Corporation, Manchester, UK) ionization source. The lock mass channel was sampled every 30 sec. The mass spectrometer was calibrated with an MS/MS spectrum of [Glu1]-Fibrinopeptide B human (Glu-Fib) solution that was delivered through the reference sprayer of the NanoLock Spray source.

### Data processing analysis, database search and quantification

For spectra processing and database search we used Progenesis QI for Proteomics software package with Apex3D, peptide 3D, and ion accounting informatics (Waters Corporation). The software loads LC-MS data, followed by chromatogram alignment and peak detection. A list of curated ions (peptides) are explored within Peptide Ion Stats by multivariate statistical methods; next, relative intensities for all ions are normalized among runs through total ion current. Finally, peptides are searched against a human proteome database (UniProt Human Reference Proteome, version 2015/11; 70,225 entries) for protein identification employing default parameters for ion accounting and quantitation 4. Protin database was reversed “on the fly” during the database queries and appended to the original database to assess the false-positive identification rate. The following parameters were considered for identifying peptides: 1) Digestion by trypsin with one missed cleavage allowed; 2) Methionine oxidation as variable modifications and carbamidomethyl (C) as fixed modification; 3) false discovery rate (FDR) lower than 1%. The protein identified with the highest score is chosen automatically as reference. Its intensity is used as a normalization factor for the relative quantification of all other proteins across the conditions analyzed (normalized abundance).

### Pathway and functional protein analysis

Proteins were analysed using the functional annotation analysis tool Panther (http://pantherdb.org/)^83^ to identify over-represented ontological groups and pathways amongst BR1 and H9 astrocyte and human adult astrocyte proteins. Using the abundant proteins found in those pathways, we explored the protein-protein interactome as an interactive representation showing the molecular relationship between molecules from the dataset based on STRING interactions with high confidence (www.string-db.org) - Search Tool for the Retrieval of Interacting Genes/Proteins^84^. The parameters used were (i) a minimum required interaction score: height confidence 0.7 and (ii) active interaction sources: textming, experiments, database, co-expression, neighbourhood, gene fusion and co-occurrence.

### Neuronal morphometry

To analyse neurite outgrowth, neuronal cells cultured either on BR1 or H9 astrocyte conditioned medium or neurobasal medium (Invitrogen) were measured using the NeuronJ plug-in in the ImageJ 1.36b software. At least ten fields were measured per well. In all cases, at least 100 neurons randomly chosen were observed per well. All neurites that emerged from neuronal soma were considered. Neurite length was analysed using 3 different methods either considering only the major process per neuron, the sum of all neurite measurements per neuron and the sum of all neurite measurements divided by the number of processes per neuron^7^.

### Statistical Analysis

Astrocytes were isolated from cerebral organoids derived from different series of organoid production (1-BR1and 2-H9), the number of differentiation experiment performed to obtain enriched astrocytes was successfully achieved in three different times (N=3). The individual assays with passage 4 astrocytes were performed three times, in triplicate, with different pool of cells, before and after freezing. Statistical analyses were performed using one-way nonparametric ANOVA coupled to Tukey’s post-test using GraphPad Prism 4.0 software, and P < 0.05 was considered statistically significant.

## Additional Information

**How to cite this article:** Dezonne, R. S. *et al*. Derivation of Functional Human Astrocytes from Cerebral Organoids. *Sci. Rep.* **7**, 45091; doi: 10.1038/srep45091 (2017).

**Publisher's note:** Springer Nature remains neutral with regard to jurisdictional claims in published maps and institutional affiliations.

## Figures and Tables

**Table 1:** Summary of molecular markers analysed in organoids derived astrocytes by immunocytochemistry.

Classification*	Markers§	BR1 astrocyte	H9 astrocyte
Neuronal	βTubulin III	−	−
Neuronal/Astroglial	NMDA receptor	+	+
Progenitor	Nestin	−	−
	Olig 2	−	−
	Foxg1	−	−
Astroglial	Vimentin	+	+
	GFAP	+	+
	GLAST	+	+
	S100β	+	+
	BLBP	+	+
	GLT1	+	+
	G. synthetase	+	+

^*^Type of neural lineage in which the specific marker is commonly found.

^§^Specific markers identified by immunocytochemistry.
